# Genotyping-by-Sequencing in *Vigna unguiculata* Landraces and Its Utility for Assessing Taxonomic Relationships

**DOI:** 10.3390/plants10030509

**Published:** 2021-03-09

**Authors:** Diana Lucia Zuluaga, Lucia Lioi, Chiara Delvento, Stefano Pavan, Gabriella Sonnante

**Affiliations:** 1 Institute of Biosciences and Bioresources (IBBR), National Research Council (CNR), via Amendola 165/A, 70126 Bari, Italy; diana.zuluaga@ibbr.cnr.it (D.L.Z.); lucia.lioi@ibbr.cnr.it (L.L.); 2 Department of Soil, Plant and Food Science, University of Bari “Aldo Moro”, via Amendola 165/A, 70126 Bari, Italy; delventochiara@gmail.com (C.D.); stefano.pavan@uniba.it (S.P.)

**Keywords:** *Vigna unguiculata*, cowpea, *Vigna* taxonomy, GBS, SNP, landraces

## Abstract

Genotyping by sequencing (GBS) was used to analyze relationships among cowpea and asparagus bean landraces from southern Italy and to assess the utility of this technology to study taxonomy in a wider panel, including *V. unguiculata* cultigroups, subspecies, and other *Vigna* species. The analysis of SNPs derived from GBS highlighted that, among the cowpea landraces, the African samples were separated from the other material, while, for the Italian landraces, a certain clustering depending on seed color/pattern was observed in the dendrogram. When examining the *V. unguiculata* species complex, a clear separation between the two groups of wild subspecies, i.e., the allogamous wild perennials and the perennial out/inbreds, could be observed, the former representing the more ancestral wild progenitors of *V. unguiculata*. The species *V. vexillata* appeared more closely related to *V. unguiculata* than to the other *Vigna* species analyzed.

## 1. Introduction

**Figure 1:**
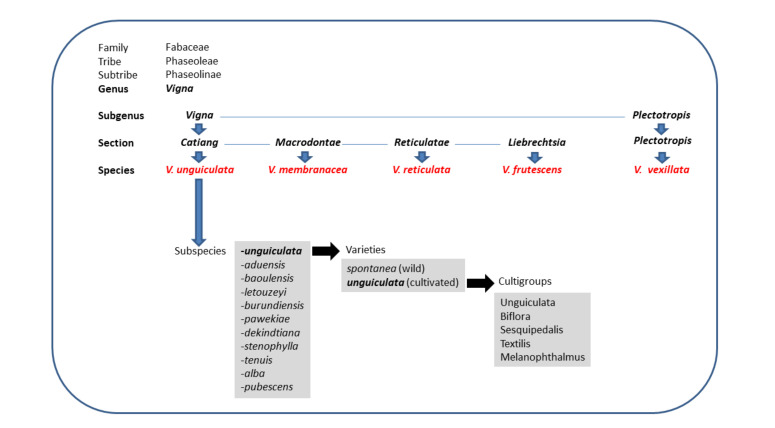
Summary of the taxonomy of the *Vigna* Savi genus (limited to the taxa considered in this study) and of the *V. unguiculata* (L.) Walp. species complex.

The genus *Vigna* Savi belongs to the botanic family of *Fabaceae*. As modified by Maréchal et al. [1], subsequently partially by Pasquet [2,3], and amended by Maxted et al. [4], the genus *Vigna* contains about 100 species distributed among six subgenera: *Vigna, Haydonia, Plectotropis, Ceratotropis, Lasiospron,* and *Sigmoidotropis*, after the relocation of the subgenus *Macrorynchus* to the genus *Wajira* [5]. The subgenus *Vigna*, or African *Vigna*, comprises six sections (*Vigna*, *Comosae*, *Macrodontae*, *Reticulatae*, *Liebrechtsia*, and *Catiang*) and 38 species; the section *Catiang* includes one of the most important food and forage legumes in the semiarid and arid tropics, the *Vigna unguiculata* (L.) Walp. species, encompassing 11 subspecies (Figure 1), 10 of which are wild. Five wild subspecies (*V. unguiculata* ssp. *aduensis*, *V. unguiculata* ssp. *baoulensis*, *V. unguiculata* ssp. *letouzeyi*, *V. unguiculata* ssp. *burundiensis*, and *V. unguiculata* ssp. *pawekiae)* are allogamous perennials, distinguished from one another based on floral traits. Five additional subspecies, *V. unguiculata* ssp. *dekindtiana*, *V. unguiculata* ssp. *stenophylla*, *V. unguiculata* ssp. *tenuis, V*. *unguiculata* ssp. *alba,* and *V. unguiculata* ssp. *pubescens*, are perennial out/inbred taxa associated with drier coastal environments. Finally, *V. unguiculata* ssp. *unguiculata* includes wild annuals, which are classified as var. *spontanea*, and cultivated forms, recognised as var. *unguiculata* [4,6]. Variety *unguiculata* is further divided in five cultigroups, based primarily on seed and pod characters: Unguiculata (cowpea or black-eyed bean) grown as a pulse and as a vegetable, Biflora (catjang) mainly used as a forage, Sesquipedalis (yardlong or asparagus bean) grown as a vegetable*,* Textilis cultivated for the fibres of its long floral peduncles [7], and Melanophthalmus [8,9] (Figure 1).

Cowpea is one of the most nutritious grain legumes containing high levels of folic acid and antioxidant and possessing free radical scavenging activities [10,11]. Additionally, cowpea has a great adaptation capacity to high temperatures and drought compared to other crop species [12]. Cowpea is the principal source of protein for people in developing countries, being mainly produced and consumed by sub-Saharan smallholder farmers. After being domesticated in Africa, this crop spread into all continents and is now commonly grown in many parts of Europe, Asia*,* and North, Central, and South America [13].

Cowpea is a diploid species with a chromosome number of 2n = 22 and an estimated genome size of 613 Mb [14]. Genomic resources, including a fragmented draft assembly, were developed for the élite breeding line IT97K-499-35 [15]. More recently, the same line was sequenced by means of single-molecule real-time sequencing, optical, and genetic mapping, to develop a new assembly, while a re-estimation of the genome size (640.6 Mbp) was obtained, based on cytometry [16]. Moreover, a 632.8 Mb assembly of the asparagus bean based on the whole genome shotgun sequencing strategy has been reported [17].

Knowledge on genetic variation is essential for developing more nutritious, productive, and resilient crop varieties inside of a breeding program, aiming for preserving global food security against the serious threat of climate change [18]. The use of molecular techniques helps in the estimation of genetic variation among genotypes [19]. In cultivated cowpea, genetic diversity has been studied using different approaches [20], including the use of molecular markers such as microsatellites [21,22,23] and single nucleotide polymorphisms (SNPs) [24,25]. Recent developments in next-generation sequencing (NGS) tools have allowed the efficient and cost-effective sequencing of plant genomes, and can be employed in plants to directly detect SNPs at a genome-wide scale [26,27]. The genotyping-by-sequencing (GBS) approach allows a rapid development of high-throughput SNPs for germplasm analysis [28,29,30,31,32]. The use of GBS has also been extended to cowpea to study, for instance, the genetic diversity among a wide collection of cowpea germplasm [25]. This study clustered genotypes in three groups and supported the hypothesis that West and East Africa represent the first domestication regions and that India is a sub-domestication area for cultivated cowpea. Additionally, the GBS approach was also used on a set of cowpea mini core lines in order to understand the underlying genetic diversity and population structure among the germplasm of this crop [33].

In the present study, we used GBS to analyze relationships among local varieties of cowpea and asparagus bean from southern Italy and to evaluate whether this technology can be useful for assessing relationships in a wider taxonomic panel including *V. unguiculata* cultigroups, subspecies, and other *Vigna* species.

## 2. Results

### 2.1. GBS Experiment and SNP Calling

The sequencing of the GBS library obtained from 49 *Vigna* accessions (Table S1) yielded about 282 million reads and 261 million good barcoded reads. Unique sequences following the barcode (tags) occurring at least three times were about 3.5 million. The total number of SNPs in the raw file was 357,862. After a quality control procedure, a variant call format (vcf) file, containing 46,658 SNPs was obtained. A smaller vcf file was also created by extracting SNP information from the 43 accessions belonging to the species *V. unguiculata* (Table S1).

### 2.2. Genetic Structure and Diversity

**Figure 2:**
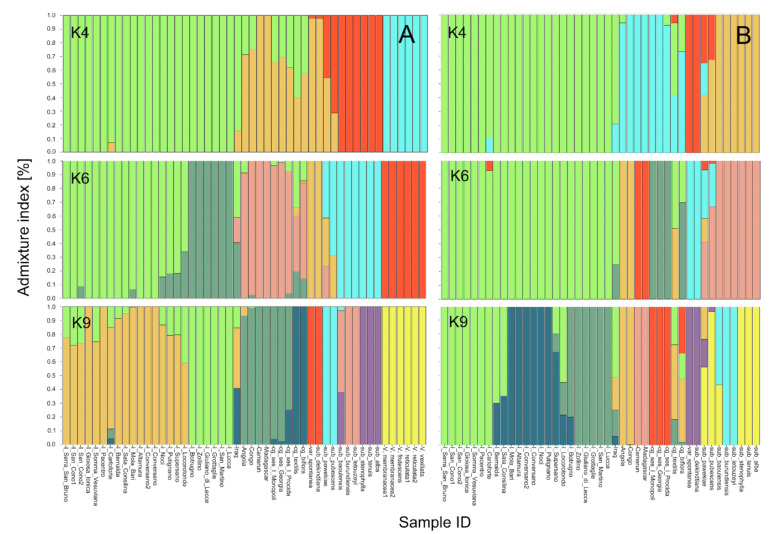
Population structure analysis of the *Vigna* germplasm used in this study. K4, K6, K9 barplots for the whole *Vigna* germplasm (**A**) and for the *V. unguiculata* accessions (**B**). Numbers on the *y*-axis indicate the estimated membership coefficient (q). Accession names are shown at the bottom of the figure. The different colors of the bars indicate the groups formed at the different K values.

In order to study the genetic structure, the parametric model implemented by the software ADMIXTURE [34] was first applied to the whole dataset (49 samples), and then uniquely to the *V. unguiculata* species (43 samples). Based on the cross-validation error test, six subpopulations (K6) were assumed to best explain the genetic structure (Figure S1A). However, since useful information can be retrieved by different levels of structure [31], we also analyzed other structures (Figure 2A, Figure S2) and considered K4, K6, and K9 as the most informative clustering arrangements, with K4 as the first level of structure, and K6 and K9 as substructuring levels (Figure 2A). Using a membership coefficient >80%, in the K4 model (Figure 2), the first group is composed of the Italian and the Iraqi cowpeas, the second one includes two African cowpeas together with subsp. *dekindtiana* and var. *spontanea*, while the other two African cowpeas and all the genotypes belonging to the different cultigroups are admixed. The third cluster embraces six subspecies (*baoulensis*, *burundiensis*, *letouzeyi*, *stenophylla*, *tenuis*, and *alba*), while subsp. *pubescens* and *pawekiae* are admixed. Finally, the fourth group includes the other *Vigna* species. Altogether, 18.37% of the material is admixed in the K4 model. In the K6 structure, the previous first group is divided into two clusters, a light green group with 16 Italian local varieties and a dark green one including Italian cowpeas with cream black-eyed seeds, whereas I-Locorotondo and Iraq are admixed (Figure 2A). The third pink group is composed of the African cowpeas together with the accessions belonging to the cultigroup Sesquipedalis, while the cultigroups *textilis* and *biflora* are admixed. Variety *spontanea* and subsp. *dekindtiana* constitute a new group (orange). The fifth (blue) and the sixth (red) groups match the third and fourth groups in the K4 model, respectively. The total of admixed samples in the K6 model is six, corresponding to 12.24%. At K9, the first group corresponds almost to the K6 first cluster, but with a higher number of admixed samples, while the second group is identical in the two models. Group 3 (dark green) matches the previous third group, except for one Sesquipedalis sample, which is admixed. The cultigroups *textilis* and *biflora* stand out as a separate group (dark blue, Figure 2A). The different subspecies form three groups, except for subsp. *dekindtiana* (included in group 5, red) and subsp. *baoulensis*, which is admixed. The percentage of admixed samples for K9 is 22.45%. In the last model analyzed, K = 10, the species *V. vexillata* separates from the other *Vigna* species (Figure S2).

Since the panel of 49 samples encompassed very distantly related germplasm material, the structure analysis was also performed considering only the 43 genotypes belonging to the species *V. unguiculata* and including 28 cowpeas: 23 Italian accessions, most of which are from the south of Italy, four African and one Iraqi accession. Models from K = 2 to K = 10 were considered (Figure S2B and Figure 2B), but the analysis was mainly focused on K4, K6, and K9 structures. At K4, the best model according to the cross validation error test (Figure S1B), the first group (green) includes all the Italian cowpeas, and the second one is composed of the African cowpeas, except for Angola (admixed) together with the three Sesquipedalis samples. Variety *spontanea* and subsp. *dekindtiana* constitute a separate group (red) as previously observed in the whole dataset (K6 in Figure 2A), and a fourth group includes six different subspecies, as in the third group of the whole dataset at K4 (Figure 2A). The K6 model shows the same first grouping as in K4, whereas the African cowpea accessions are divided in two groups, Madagascar-Camerun (red) and Angola-Congo (orange, Figure 2B), and a new cluster includes the three accessions of cultigroup Sesquipedalis (dark green). Groups 5 and 6 correspond to groups 3 and 4 in the K4 model, respectively. In K8 (Figure S2B), it is worth noting that the Italian cowpeas are divided into two groups (orange and pink), almost identical to those observed in K6 for the whole dataset (Figure 2A). In K9, the cowpea orange group shown in K8 is further divided in two groups, one composed of accessions from the Apulia region with mostly black full coat seeds (dark blue cluster), the other including material from central Italy and southern regions other than Apulia, with cream brown-eyed seeds or light brown full coat (light green cluster) (Figure 2B and Figure S3). Groups 4, 5, 6, and 7 in K9 correspond to groups 2, 3, 4, and 5 in K6, respectively, while the different subspecies are arranged in two groups and two admixed samples.

Based on K4 clustering for the whole dataset, some diversity parameters were calculated (Table S2). We chose this K4 model, since it was associated with one of the lowest cross validation errors for which the Italian cowpea accession clustered together. The first group observed, composed of the Italian cowpeas and the accession from Iraq, showed the lowest values for GD (Nei’s genetic diversity), PIC (polymorphic information content), He (expected heterozygosity), and Fi (inbreeding coefficient) compared to the other three groups, whose values for the same parameters were similar. On the other hand, Ho (observed heterozygosity) for group 1 was lower than in other groups. The lowest pairwise Fst value was observed when comparing group 2 (some African cowpeas with the wild cowpea progenitor) with group 3 (six different *V. unguiculata* subspecies), whereas the highest value was between K1 (Italian and Iraqi cowpea) and K4 (*Vigna* species other than *V. unguiculata*). Nei’s genetic distance ranged between 0.062 (K1–K2) and 1.095 (K1–K4).

### 2.3. Genetic Relationships among Accessions

**Figure 3:**
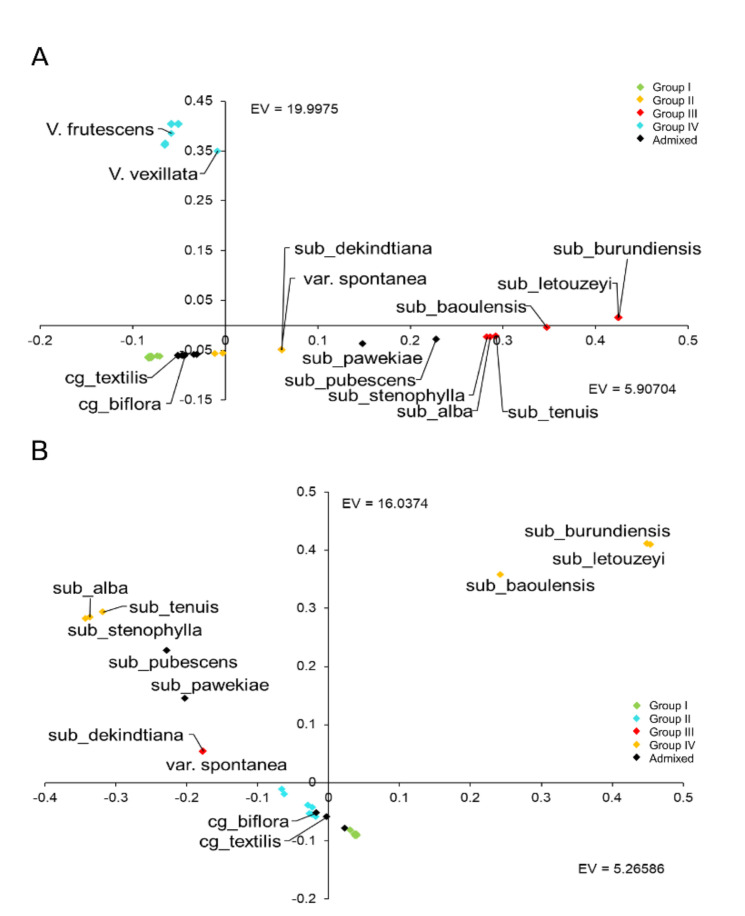
Principal Component Analysis (PCA). Diagram of the first two axes from a PCA of 49 *Vigna* accessions (**A**) and 43 *V. unguiculata* samples (**B**). The different colors correspond to the groups formed in the population structure analysis at the K4 model.

A Principal Component Analysis (PCA) was performed in order to assess genetic relationships among the *Vigna* samples (Figure 3). In the PCA for the whole dataset (Figure 3A), the first principal component accounts for 50.40% of the overall variability, while the second one explains 14.89% of it. The plot shows a spatial distribution of the germplasm material, with accessions grouping similarly to what was observed in K4 of the structure analysis. A compact group is composed of the cowpea accessions (green), except for the accessions from Africa—Congo and Angola (black, admixed), Cameroon and Madagascar (orange, group 2)—which are separated from the other cowpeas. In between the Italian and the African cowpeas, there are the other cultigroups of *V. unguiculata* subsp. *unguiculata*, i.e., Sesquipedalis, Textilis and Biflora (black, admixed). Moving towards the right side of the graph, the wild var. *spontanea* is close to subsp. *dekindtiana* (orange, as the cowpea from Cameroon and Madagascar), and then all the other subspecies of *V. unguiculata* can be found (group 3 pink, except for subsp. *pawekiae* and *pubescens*, black). The other *Vigna* species considered are placed in the upper part of the graph, on the left-hand side (blue, group 4), with *V. vexillata* being quite isolated. All of them are very distant from *V. unguiculata*.

A PCA plot was also obtained using only the *V. unguiculata* germplasm (Figure 3B), where the first principal component explains 45.34% of the overall variability, while the second one accounts for 14.89% of it. For some aspects, the distribution of these samples is similar to the one observed for the whole data set. The Italian cowpea material (green, group 1) is clustered together while group 2 (blue), which includes most African cowpeas together with the cultigroup Sesquipedalis, is somewhat more scattered. Variety *spontanea* and subsp. *dekindtiana* are close together (orange, group 3), as in the previous graph. The other *V. unguiculata* subspecies are quite scattered, showing that subsp. *baoulensis*, subsp. *burundiensis*, and subsp. *letouzeyi* are distant from the other subspecies and close to one another, although, at K4 in the structure analysis, they are clustered together with subsp. *alba*, *tenuis*, and *stenophylla*. A separation between these two groups of subspecies can be observed in the substructure at K9 (Figure 3A,B). Additionally, to attain a deeper insight, a PCA plot considering the cowpea and the cultigroup Sesquipedalis accessions was also obtained (Figure S4). In this chart, the Cameroon and Madagascar accessions are placed in the left upper part of the graph while Angola and Congo are located more in the central part of the figure. On the other hand, the three Sesquipedalis accessions are placed on the right side of the graph. The Italian cowpeas are all very close in the graph, with the exception of the I_Carloforte accession and the Iraqi accession, which are more distant from the other Italian germplasm (Figure S4). It is possible to differentiate a group of Italian cowpeas (I_Botrugno, I_Grottaglie, I_Lucca, I_Zolino, I_Giuliano_di Lecce) that are grouped together separately from the other cowpeas.

**Figure 4:**
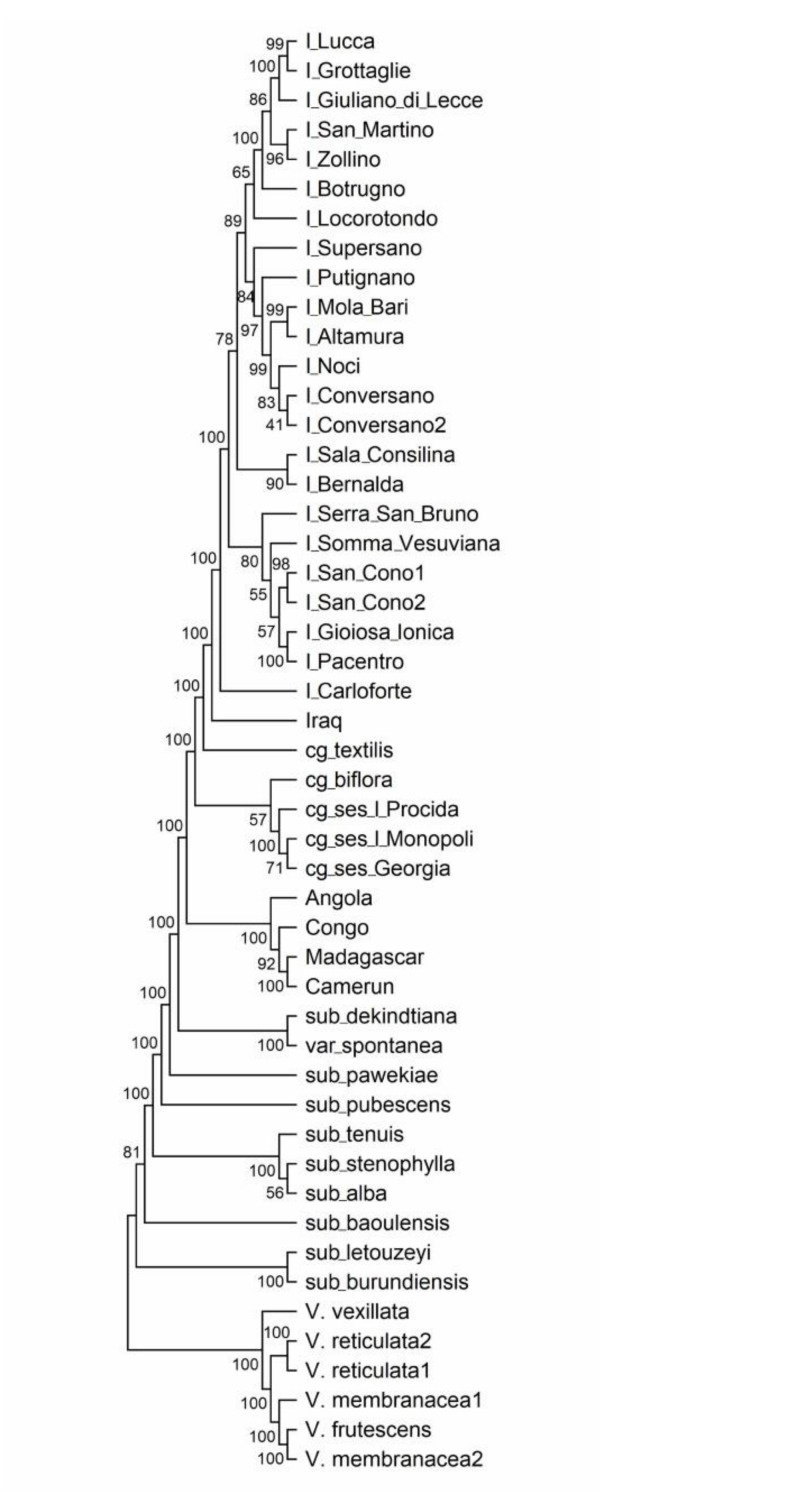
Neighbor-Joining tree. Neighbor-Joining tree obtained from SNP data on the whole *Vigna* dataset. Numbers on tree branches indicate bootstrap values.

The Neighbor-Joining (NJ) clustering analysis performed using the whole dataset produced a tree that highlights two main branches, one including all the samples belonging to the species *V. unguiculata*, and the other containing the species *V. vexillata*, *V. reticulata*, *V. membranacea*, and *V. frutescens* (Figure 4). The latter four species show relatively low genetic distances among one another, while the highest distances can be found between each of them and the *V. unguiculata* samples, with a lower value between *V. vexillata* and *V. unguiculata* (Table S3). Subsp. *dekindtiana* clusters together with subsp. *unguiculata* var. *spontanea*, separately from the other subsp. *unguiculata* samples. The following group, including the African cowpea genotypes, is more basal compared to the cultigroups other than Unguiculata. In fact, samples from the other cultigroups have a slightly lower genetic distance to the Italian/Iraqi cowpeas than to the African cowpea (Table S3). Apart from I_Carloforte, the Italian cowpea material appears subdivided into two main branches. The larger cluster contains two main groups, the upper one, constituted of accessions with cream black-eyed seeds (Figure S3), the lower group including mainly black full coat seeded accessions. The second branch of the Italian material is made of accessions with light brown full coat seeds or brown-eyed seeds (Figure S3).

## 3. Discussion

In the present work, the GBS technology was used to assess genetic relationships among cowpea and asparagus bean landraces, mainly originating from the Apulia region and Southern Italy, and to study taxonomic relationships within the *V. unguiculata* species complex and between this and other *Vigna* species. Although only one sample was generally considered for *Vigna* taxa other than cowpea and asparagus bean, meaningful results were obtained by the study of genetic variation. For instance, as highlighted by both PCA and the NJ clustering, *V. vexillata* appears to be the most isolated taxon. In fact, *V. vexillata* belongs to the subgenus *Plectotropis*, while all the other samples analyzed here are included in the subgenus *Vigna*. However, when considering the genetic distances, our data reveal that *V. vexillata* is more closely related to *V. unguiculata* than to the other *Vigna* species analyzed. Although some authors regarded *V. vexillata* as an intermediate species between African and Asian *Vigna* [35], molecular analyses suggested that *V. vexillata* was closer to the African subgenus *Vigna* section *Catiang* than to Asian species [36]. Moreover, *V. unguiculata* was found to be genetically closer to *V. vexillata* than to other species of the subgenus *Vigna*, section *Catiang* [37,38]. The highly pubescent species *V. vexillata* has drawn the attention of researchers since it holds genes for resistance to cowpea pests, and quantitative trait loci (QTLs) mapping for resistance to the insect *Callosobruchus* species has been performed [39]. In addition, successful hybridization between this species and cowpea has been described [40].

Within the species *V. unguiculata*, three groups of subspecies have been identified according to the breeding system, namely the allogamous wild perennials, the perennial out/inbreds, and the annual inbreds (subsp. *unguiculata*). The structural analysis with just the *V. unguiculata* species showed that, at K5 and at K9, the subspecies different from subsp. *unguiculata* are split into two groups, one including *burundiensis*, *baoulensis* (admixed at K9), and *letouzeyi*, and the second group containing *alba*, *tenuis*, *stenophylla*, and *pubescens*. In these models, there is a separation of the outbreeding subspecies in the first group, and the out-inbreds in the second one. In the PCA analyses, the two groups, allogamous and out/inbreeding subspecies, are differently positioned, and, in the NJ tree, the former group occupies a more basal position compared to the latter one. Morphological and molecular data obtained by Pasquet [41] revealed that, in the allogamous wild perennials, floral characters separate the subspecies, while the taxa of the perennial out/inbreds display strong morphological features such as pubescence, seed size, or leaf shape. Additionally, geographically, all the allogamous subspecies (and the outcrossing parts of the out/inbreds) are limited to Guinean or highland areas, whereas perennial inbreds are found in larger and drier areas with coastal distribution. Subspecies *pawekiae*, although being outcrossing, shows an admixed genetic background in the K5 and K9 models and is found to be quite isolated from the other subspecies, but closer to the out/inbreeding group in the PCA and in the NJ tree. Subspecies *pawekiae* is very widely distributed in Africa [41], and perhaps this fact could influence the genetic differences observed in this work compared to the other allogamous subspecies, also considering that here only one genotype was analyzed. The above findings, together with the genetic distances observed for our GBS data, seem to confirm previous studies suggesting that the allogamous wild perennial subspecies are more primitive and therefore could represent the first step in the wild cowpea evolution; the second step would have led to the diversification of the perennial out-inbreds, which could have evolved more recently [6].

Among the subspecies analyzed, this study reveals that subsp. *dekindtiana* is the entity more closely related to cowpea, showing a considerably lower distance compared to the other subspecies. Surprisingly, the subsp. *dekindtiana* sample displayed genetic distances to all other samples identical or comparable to the distances observed for the wild subsp. *unguiculata* var. *spontanea* (Table S3). The specimen we analyzed was obtained from IPK genebank in Gatersleben (Germany) and classified as *V. unguiculata* subsp. *dekindtiana*. The taxonomy of the genus *Vigna* in general and of the species complex *V. unguiculata* in particular has undergone several revisions in the last decades of 1900. Verdcourt [42] and Maréchal et al. [1] used the term “*dekindtiana*” (subsp. *dekindtiana* or subsp. *dekindtiana* var. *dekindtiana*, respectively) to indicate all the non-austral non-pubescent spontaneous forms with short-lobe calyx. This definition was too wide and later Pasquet [2] derived different taxa from the previous “*dekindtiana*”, including var. *spontanea*, which is considered the progenitor of cowpea [6,43]. Therefore, since the two samples of subsp. *dekindtiana* and var. *spontanea* we analyzed were practically genetically identical, we checked in the IPK records and found that this specimen was obtained from Meise Botanical Garden (Belgium) in 1987. While the Meise Botanical Garden updated the nomenclature of its *Vigna* material, IPK kept the previous nomenclature, and therefore this sample is considered as *V. unguiculata* subsp. *unguiculata* var. *spontanea* for the former, and *V. unguiculata* subsp. *dekindtiana* for the latter.

The African cowpeas appear well separated from the Italian landraces and are placed in a more basal position in the tree, compared to the cultigroups Textilis, Biflora, and Sesquipedalis, due to a higher genetic distance to the other cowpea material, especially for the accessions from Cameroon and Madagascar. In a previous study based on AFLP markers, *V. unguiculata* accessions from different cultigroups were not clearly separated, but mixed up in the tree [44]. In an analysis founded on phenotypic data, African cowpea material was far apart from Spanish and Portuguese germplasm [20], while an SNP analysis of a wide set of cowpea landraces revealed that accessions from Europe were more related to those from western than from eastern Africa [24].

Our genetic analyses highlighted a certain grouping of the Italian cowpeas according to the seed color/pattern, suggesting a possible common genetic background for the material sharing similar patterns, although the number of accessions investigated here is too limited to perform an association mapping study. A wide variation in seed coat color and pattern can be observed in cowpea germplasm, and this character is an economically important trait, since related to consumers’ preferences, especially where dry seeds are used [45,46,47,48,49]. Seed color can vary from cream to light brown, reddish-brown, or black with various patterns including different eye shapes and sizes, and forms including speckling, blotching, marbling, or full coat. Cowpea seed coat traits have been investigated since the early 1900s [50,51,52], when genetic factors responsible for color expression were identified: Color Factor (C); Watson (W), Holstein-1 (H-1), Holstein-2 (H-2). Later, a three-locus system was produced [53,54,55], and recently various seed coat pattern traits were mapped to three loci concurrent with the C, W, and H factors, and candidate genes related to the regulation of the later steps of the flavonoid biosynthesis were identified [56].

## 4. Materials and Methods

### 4.1. Plant Material

A group of 49 accessions of *Vigna* were analyzed in the present study. Most material was constituted of cowpea landraces (12 from Apulia region, southern Italy; 11 from other Italian regions; 5 from other countries; Table S1). Three accessions belonged to the cultigroup Sesquipedalis, one to the cultigroup Textilis and another one to the cultigroup Biflora. One wild accession of *V. unguiculata* subsp. *unguiculata*, var. *spontanea*, was also included. Moreover, one accession of each one of the *V. unguiculata* subspecies *baoulensis*, *burundiensis*, *letouzeyi*, *pawekiae*, *dekindtiana*, *stenophylla*, *tenuis*, *alba* and *pubescens* was added. Additionally, other *Vigna* species were included in this study, three belonging to the subgenus *Vigna*: *V. frutescens* (section *Liebrechtsia*, one accession), *V. membranacea* (section *Macrodontae*, two accessions), *V. reticulata* (section *Reticulatae*, two accessions), and one species, *V. vexillata*, belonging to the subgenus *Plectotropis*.

Cowpea and asparagus bean landraces from the Apulia region were recently collected from farmers’ fields (PSR_BIOD code); the other material was obtained from the Institute of Biosciences and Bioresources genebank (MG code), from Meise Botanic Garden, Belgium (NI codes), or from the The Leibniz Institute of Plant Genetics and Crop Plant Research (IPK), Germany (VIG codes).

### 4.2. GBS Assay and SNP Filtering

Genomic DNA from *Vigna* young leaves was isolated as in Curci et al. [57]. After measuring DNA concentration, equal amounts of DNA were sent to the Genomic diversity facility of the Cornell University (Ithaca, NY, USA) (http://www.biotech.cornell.edu/ accessed on 15 May 2019) for library preparation using the enzyme *Ape*KI and sequencing by means of a HiSeq2000 Illumina machine in high output mode (100 bp reads), as a single lane containing an empty, negative control sample. The sequencing reads were examined for the barcodes matching 100% with the expected bases remnant of the enzyme restriction site. The barcode containing reads were organized, de-multiplexed, and trimmed to first 64 bases starting from the enzyme cut site. Then, the “N” containing reads within the initial 64 bases were excluded.

In order to perform the SNP call and generate a variant call format (vcf) file, the Discovery TASSEL-GBS pipeline [58] was used together with the Cowpea_Genome_0.03 sequence, kindly provided by Timothy Close at University of California, Riverside, CA, USA. Biallelic SNPs were filtered based on a call rate higher than 80%, a minor allele frequency (MAF) higher than 5% and an inbreeding coefficient higher than 80% using TASSEL v5.2.20 [59].

GBS short reads were submitted to the SRA NCBI public database under the BioProject number PRJNA689726. The SNPs analyzed here are included in Table S4.

### 4.3. Genetic Structure Analysis, Population Genetic Diversity, and Relationships

The population structure of the *Vigna* germplasm under study was analyzed using the ADMIXTURE software [34]. The best K value was calculated based on the lower 10 fold cross validation test. Barplots per each K value were graphed using *StructuRly* 0.1.0 program (https://nicocriscuolo.shinyapps.io/StructuRly/ (21 May 2020). Genetic relationships were evaluated among all *Vigna* samples and within the *V. unguiculata* genotypes, through principal component analyses (PCA), which were performed using SVS v.8.4.0 (Golden Helix Inc., Bozeman, MT, USA). Genetic distances, based on the p-distance method [60], were used to construct a Neighbor-Joining tree [61], with the MEGA X package [62].

Based on the K4 structure model for the whole dataset, the following parameters were calculated for each group: GD (Nei’s genetic diversity), PIC (polymorphic information index), Ho (observed heterozygosity), He (expected heterozygosity), Fi (inbreeding coefficient), pairwise Nei’s standard genetic distance, and pairwise Fst analysis, using snpReady package in R [63].

## 5. Conclusions

The analysis of SNPs, obtained by means of a reduced representation of the genome through GBS technology, provided useful information for the analysis of cowpea landraces, highlighting patterns of geographical distribution and a possible grouping related to seed color/pattern. Genome-wide SNPs have proven useful for corroborating taxonomic relationships within the species *V. unguiculata* and between this species and other *Vigna* species.

## Data Availability

The short reads presented in this study are openly available in the SRA NCBI public database under the BioPro-ject number PRJNA689726. The SNPs analyzed are included in Table S4.

## Supplementary Materials

The following are available online at https://www.mdpi.com/2223-7747/10/3/509/s1, Table S1: List of the germplasm material analyzed in this study and relevant information., Table S2: Population genetic indexes for K1, K2, K3 and K4 using groups generated by the K4 model in the structure analysis: Nei’s genetic diversity (GD), Polymorphic information content (PIC), Observed heterozygosity (Ho), Expected heterozygosity (He), Inbreeding coefficient (Fi), Effective populational size (Ne), Pairwise Fst matrix between groups and Pairwise Nei’s genetic distance matrix between groups., Table S3: Matrix of the pair-wise genetic distances calculated according to the p-distance method (Nei and Kumar 2000); Table S4: SNP markers analyzed in the present study; Figure S1: Cross-validation (CV) error estimates on each K tested on 49 (A) and 43 (B) *Vigna* samples., Figure S2: Population structure analysis of the *Vigna* germplasm used in this study. K2, K3, K5, K7, K8, and K10 barplots for the whole *Vigna* germplasm (A) and the *V. unguiculata* accessions (B). Numbers on the *y*-axis indicate the estimated membership coefficient (q). Accession names are showed at the bottom of the figure. The different colors of the bars indicate the groups formed at the different K values., Figure S3: Cowpea and asparagus bean landrace seeds collected in Italy and an accession from Iraq. For germplasm codes refer to Table S1. Figure S4: Principal Component Analysis (PCA). Diagram of the first two axes from a PCA of cowpea and cultigroup Sesquipedalis (A), and Italian and Iraqi cowpeas (B).
