# Supplementary material for: Genotyping-by-Sequencing in Vigna unguiculata Landraces and Its Utility for Assessing Taxonomic Relationships
Source: Plants (Basel). 2021 Mar 9;10(3):509. doi: 10.3390/plants10030509 (PMC8001400; doi:10.3390/plants10030509)
Supplement: Supplementary file 1 [file plants-10-00509-s001.zip › plants-10-00509-s001/Figure S1.docx]

**Figure S1.** Cross-validation (CV) error estimates on each K tested on 49 (A) and 43 (B) *Vigna* samples.

**B**

**A**
