# Supplementary material for: Genotyping-by-Sequencing in Vigna unguiculata Landraces and Its Utility for Assessing Taxonomic Relationships
Source: Plants (Basel). 2021 Mar 9;10(3):509. doi: 10.3390/plants10030509 (PMC8001400; doi:10.3390/plants10030509)
Supplement: Supplementary file 1 [file plants-10-00509-s001.zip › plants-10-00509-s001/Figure S2.pptx]

## Slide 1
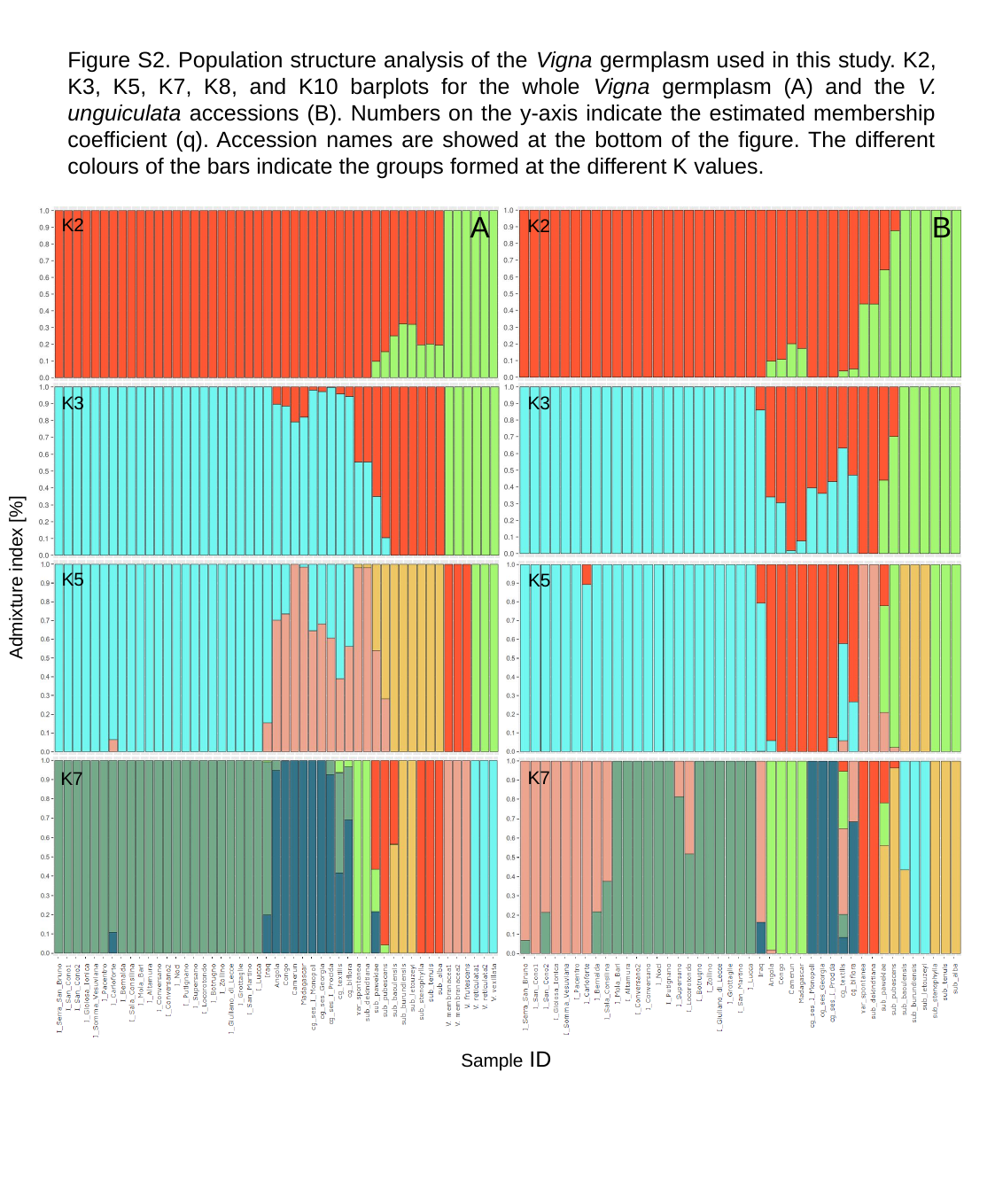

Figure S2. Population structure analysis of the Vigna germplasm used in this study. K2, K3, K5, K7, K8, and K10 barplots for the whole Vigna germplasm (A) and the V. unguiculata accessions (B). Numbers on the y-axis indicate the estimated membership coefficient (q). Accession names are showed at the bottom of the figure. The different colours of the bars indicate the groups formed at the different K values.
A
B
K2
K2
K3
K3
Admixture index [%]
K5
K5
K7
K7
Sample ID

## Slide 2
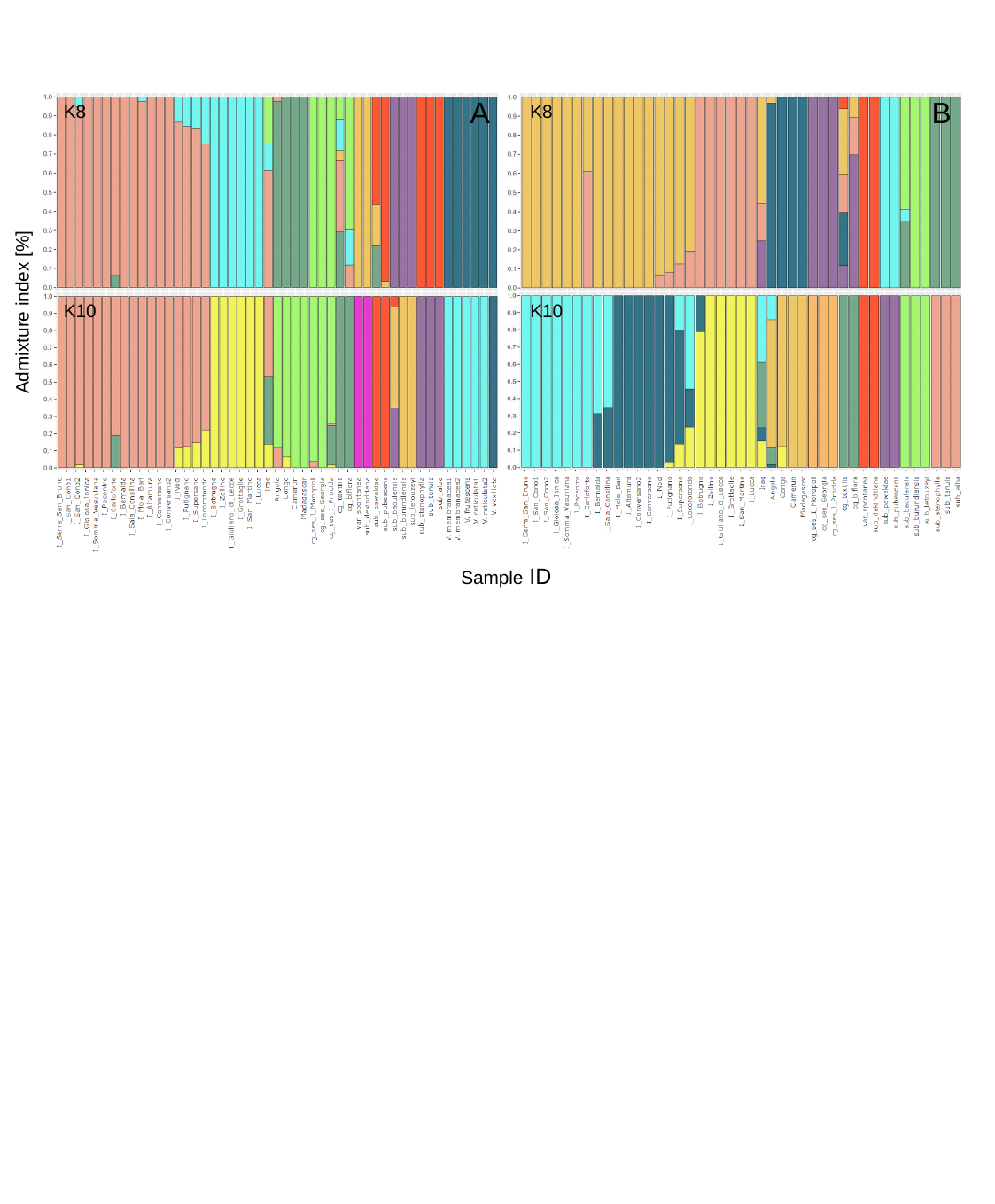

A
B
K8
K8
Admixture index [%]
K10
K10
Sample ID
