# Supplementary material for: Genotyping-by-Sequencing in Vigna unguiculata Landraces and Its Utility for Assessing Taxonomic Relationships
Source: Plants (Basel). 2021 Mar 9;10(3):509. doi: 10.3390/plants10030509 (PMC8001400; doi:10.3390/plants10030509)
Supplement: Supplementary file 1 [file plants-10-00509-s001.zip › plants-10-00509-s001/Figure S3.docx]

**Figure S3**. Cowpea and asparagus bean landrace seeds collected in Italy and an accession from Iraq. For germplasm codes refer to Table S1.

| 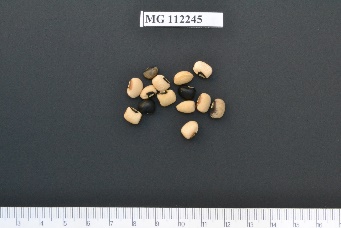 | 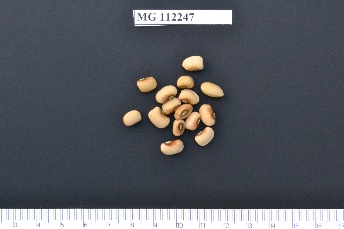 | 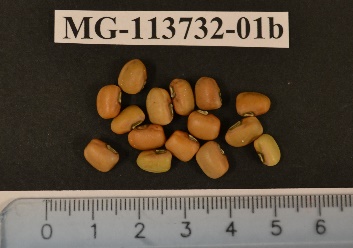 | 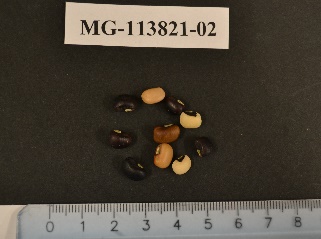 |
| --- | --- | --- | --- |
| I_Serra_San_Bruno | I_San_Cono1 | I_Gioiosa_Ionica | I_Somma_Vesuviana |
|  |  |  |  |
| 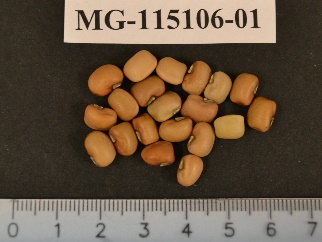 | 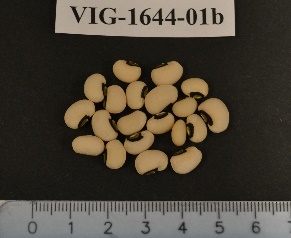 | 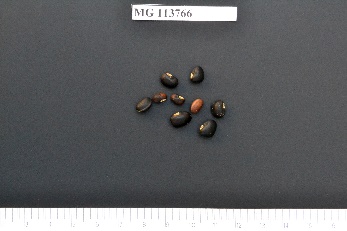 | 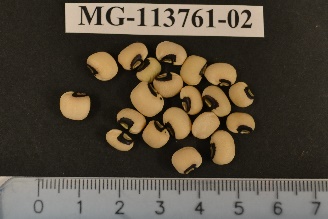 |
| I_Pacentro | I_Carloforte | I_Bernalda | I_Sala_Consilina |
|  |  |  |  |
| 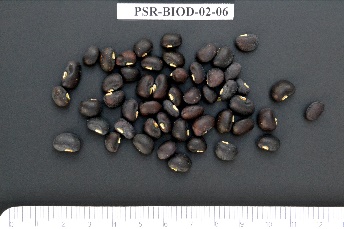 | 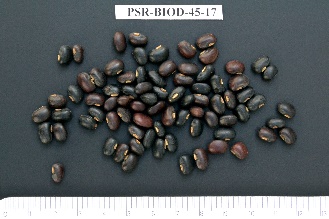 | 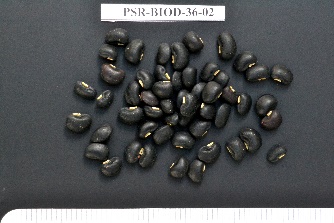 | 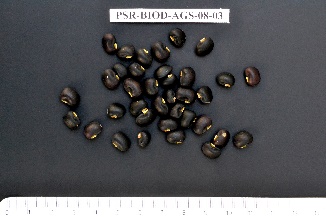 |
| I_Mola_Bari | I_Altamura | I_Conversano | I_Conversano2 |
|  |  |  |  |
| 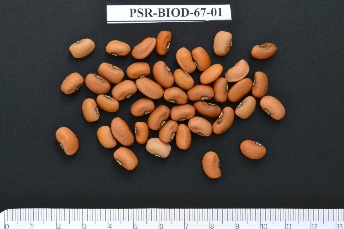 | 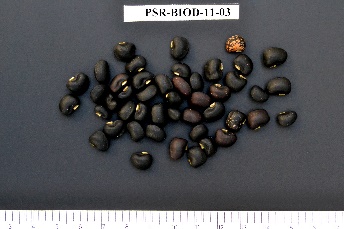 | 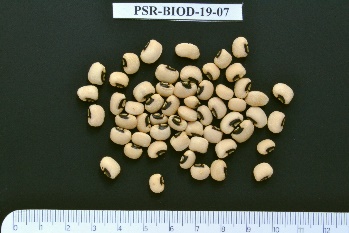 | 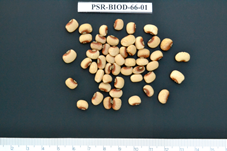 |
| I_Noci | I_Putignano | I_Supersano | I_Locorotondo |
|  |  |  |  |
| 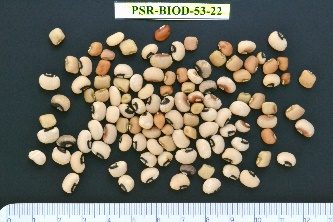 | 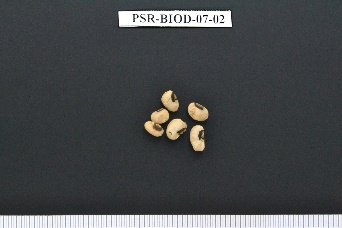 | 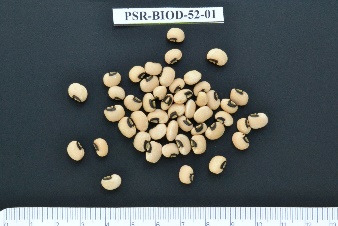 | 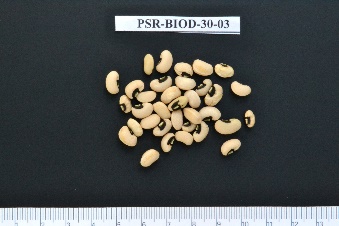 |
| I_Botrugno | I_Zollino | I_Giuliano_di_Lecce | I_Grottaglie |
|  |  |  |  |
| 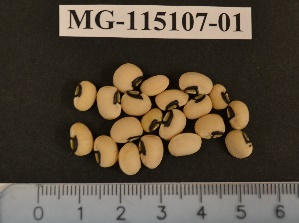 | **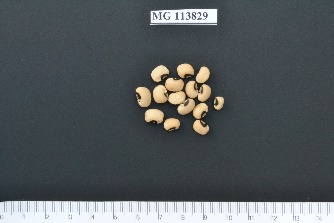** | **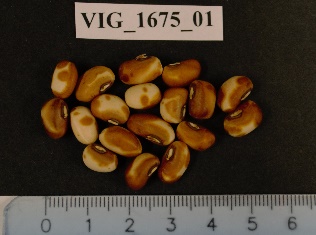** | **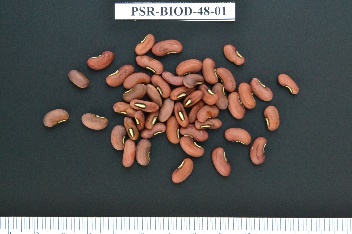** |
| I_San_Martino | I_Lucca | Iraq | cg_ses_I_Monopoli |
