# Supplementary material for: Genotyping-by-Sequencing in Vigna unguiculata Landraces and Its Utility for Assessing Taxonomic Relationships
Source: Plants (Basel). 2021 Mar 9;10(3):509. doi: 10.3390/plants10030509 (PMC8001400; doi:10.3390/plants10030509)
Supplement: Supplementary file 1 [file plants-10-00509-s001.zip › plants-10-00509-s001/Figure S4.pptx]

## Slide 1
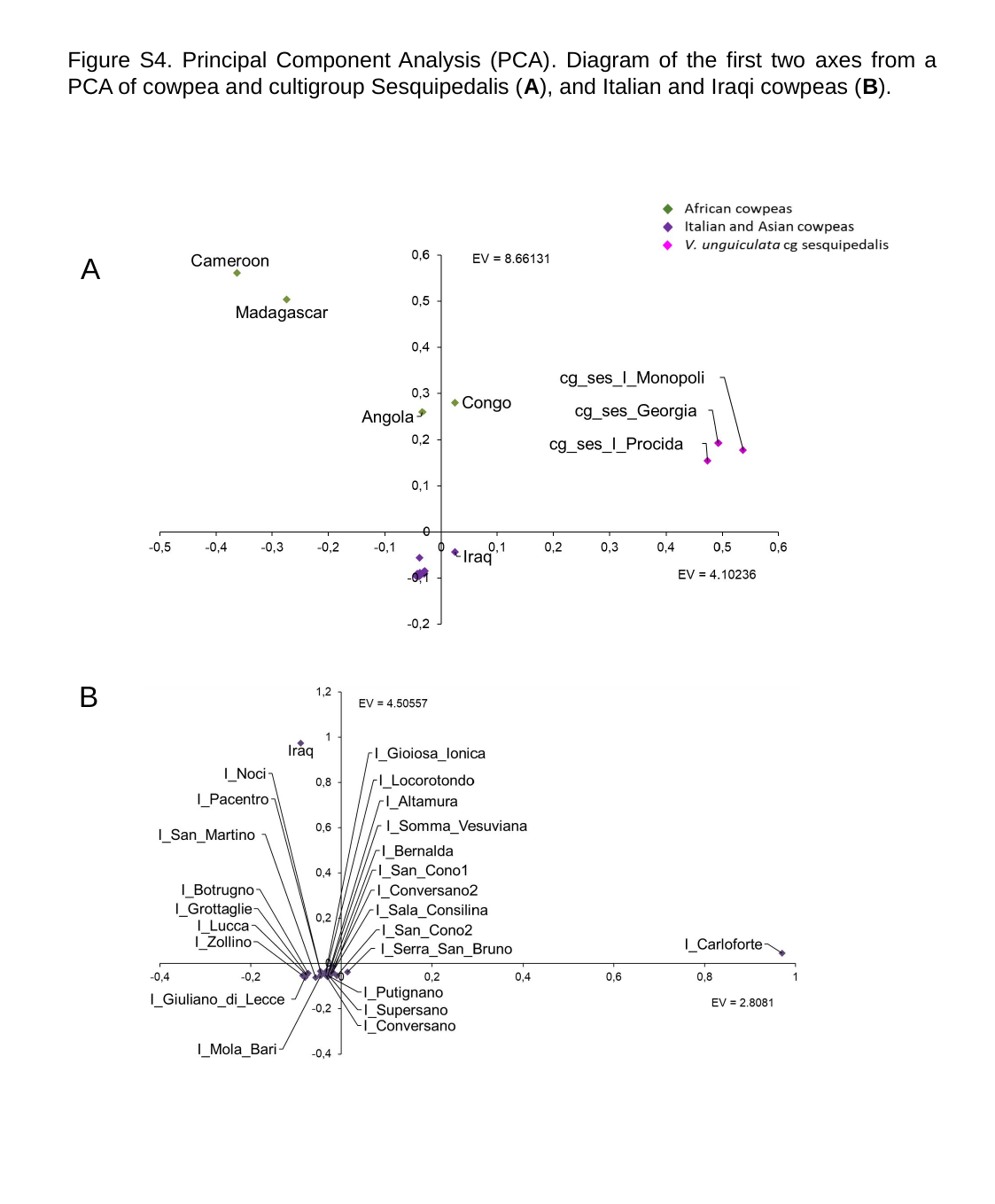

Figure S4. Principal Component Analysis (PCA). Diagram of the first two axes from a PCA of cowpea and cultigroup Sesquipedalis (A), and Italian and Iraqi cowpeas (B).
A
B
